# Bowel urgency in inflammatory bowel disease: A concept analysis

**DOI:** 10.1093/ibd/izag018

**Published:** 2026-02-25

**Authors:** Daniele Napolitano, Mattia Bozzetti, Valentina Vanzi, Alessio Lo Cascio, Ivan Capobianco, Antonio Gasbarrini, Loris Riccardo Lopetuso, Franco Scaldaferri

**Affiliations:** CEMAD, Fondazione Policlinico Universitario A. Gemelli IRCCS, 00168 Rome, Italy; Direction of Health Professions, ASST Cremona, 26100 Cremona, Italy; Center of Excellence for Nursing Scholarship, Rome, Italy; Direction of Health Professions, La Maddalena Cancer Center, 90146 Palermo, Italy; CEMAD, Fondazione Policlinico Universitario A. Gemelli IRCCS, 00168 Rome, Italy; CEMAD, Fondazione Policlinico Universitario A. Gemelli IRCCS, 00168 Rome, Italy; Dipartimento Universitario di Medicina e Chirurgia Traslazionale, Università Cattolica del Sacro Cuore, 00168 Rome, Italy; CEMAD, Fondazione Policlinico Universitario A. Gemelli IRCCS, 00168 Rome, Italy; Dipartimento Universitario di Medicina e Chirurgia Traslazionale, Università Cattolica del Sacro Cuore, 00168 Rome, Italy; Department of Life Science, Health, and Health Professions, Link Campus University, 00165 Rome, Italy; CEMAD, Fondazione Policlinico Universitario A. Gemelli IRCCS, 00168 Rome, Italy; Dipartimento Universitario di Medicina e Chirurgia Traslazionale, Università Cattolica del Sacro Cuore, 00168 Rome, Italy

**Keywords:** bowel urgency, inflammatory bowel disease, concept analysis, patient-reported outcomes, clinical assessment

## Abstract

**Background:**

Bowel urgency is a distressing and often underrecognized symptom of inflammatory bowel disease (IBD). It represents a sudden and compelling need to defecate that is difficult to defer and strongly affects patients’ quality of life. Despite its clinical importance, the concept of bowel urgency remains poorly characterized and inconsistently measured across studies.

**Aims:**

This review aims to clarify the conceptual boundaries of bowel urgency, summarize recent clinical and mechanistic evidence, and provide a framework to guide its assessment and management in clinical practice and research.

**Methods:**

A narrative concept analysis was conducted using Walker and Avant’s 8-step method. Evidence from clinical, physiological, and patient-reported outcome studies published between 2016 and 2025 was integrated across major databases (MEDLINE, PsycInfo, Scopus, Web of Science) to identify defining attributes, antecedents, and consequences of bowel urgency in IBD.

**Results:**

Seven defining attributes were identified: sudden onset, perceived uncontrollability, compressed time to toilet, fear or risk of incontinence, anticipatory anxiety, behavioral planning or avoidance, and persistence despite inflammatory quiescence. Biological antecedents include rectal inflammation, hypersensitivity, and altered pelvic floor function, while psychosocial factors such as vigilance and anxiety contribute to chronicity. Consequences extend from emotional distress and reduced social participation to increased healthcare utilization. Current tools, including the Urgency Numeric Rating Scale, capture intensity but fail to reflect multidimensional impact.

**Conclusions:**

Bowel urgency is a multidimensional clinical construct with physiological, psychological, and behavioral components. Its systematic assessment should become a routine element of IBD care and a standardized endpoint in clinical trials. Developing and validating a multidimensional, IBD-specific urgency measure would bridge symptom monitoring and patient-centered outcomes.

## Graphical abstract

**Graphical Abstract:**
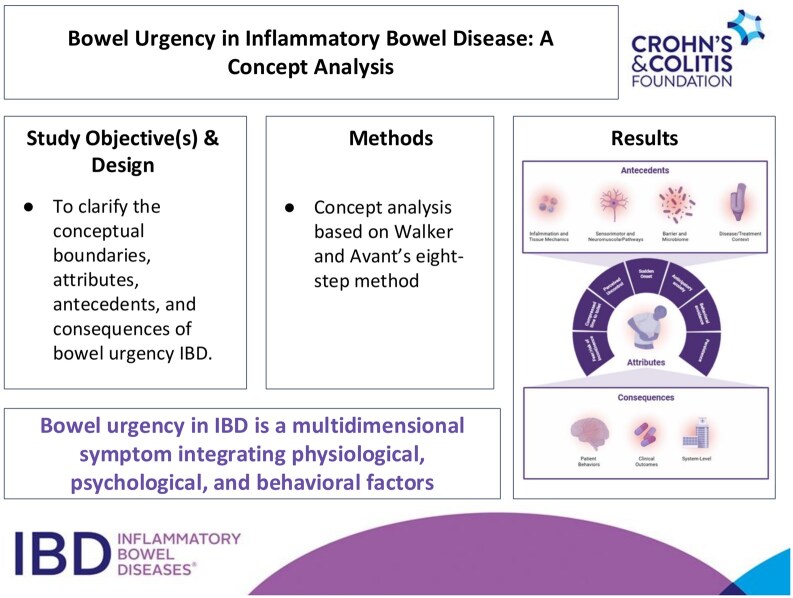

Key Messages *What is already known?*

Bowel urgency is a common and distressing symptom in ulcerative colitis and Crohn’s disease, yet it is often underestimated and insufficiently captured by existing clinical indices or single-item patient-reported measures.

*What is new here?*

This concept analysis defines 7 key attributes of bowel urgency and identifies biological and psychosocial antecedents that sustain the symptom even in remission, proposing a multidimensional framework for future measurement and intervention.

*How can this study help patient care?*

Clarifying the multidimensional nature of bowel urgency can improve clinical assessment, patient communication, and therapeutic targeting, promoting the inclusion of urgency as a standardized, patient-centered endpoint in both everyday care and clinical trials.

## Introduction

Bowel urgency (also “rectal urgency” or “fecal urgency”) is defined as a sudden and compelling need to defecate, often perceived as uncontrollable and potentially associated with fecal leakage.^1–3^ However, this definition reflects a rather monodimensional and physiopathological perspective, primarily focused on the descriptive and symptomatic aspects of the phenomenon. Bowel urgency is a complex, multidimensional concept encompassing physical, psychological, and social dimensions. It is a cardinal symptom of inflammatory bowel disease (IBD), mainly including ulcerative colitis (UC) and Crohn’s disease (CD), which are characterized by a chronic-relapsing course and a substantial impact on quality of life.^4–8^ Despite its clinical relevance, bowel urgency has long been underestimated in medical practice and rarely considered as a primary outcome in clinical trials.^9–13^ Among the most debilitating symptoms of IBD, bowel urgency is one of the main determinants of disability and healthcare costs, yet it remains surprisingly underrated.^14–17^

Evidence from the early 2010s documented, for the first time, that fecal urgency and incontinence can persist even in quiescent disease conditions and exert a substantial emotional, social, and functional impact, establishing the basis for much of the subsequent literature on this symptom.^18–20^

Epidemiological evidence shows that over 80% of patients with UC experience episodes of urgency during active phases of disease and that this symptom can also persist during remission.^1^^,^^2^^,^^11^ Multicenter studies have documented that 17% to 26% of patients with UC and 13% to 17% of those with CD report persistent urgency despite conventional treatments or targeted therapies.^4^^,^^15^ Even in clinical remission, the prevalence remains high: in a Swiss ­survey, 65.9% of patients with UC and 68.5% of those with CD reported significant urgency.^11^ These data indicate that urgency is not simply a marker of acute inflammation, but also a chronic symptom reflecting more complex pathophysiological alterations, including rectal hypersensitivity, muscle spasms, and submucosal fibrosis.^2^

The impact of bowel urgency on daily life is substantial. Several studies have documented independent associations with reduced work productivity, social isolation, mood disorders, and anxiety.^6^^,^^14^ The analysis conducted in the IBD Partners registry showed that increasing levels of urgency (“hurry,” “immediately,” “incontinence”) proportionally increase the risk of depression, anxiety, fatigue, and limitations in social participation, as well as the likelihood of hospitalization, corticosteroid use, and colectomy within 12 months.^6^^,^^21–23^ Such evidence indicates that urgency is not only an indicator of disease activity but also a predictor of adverse clinical outcomes.^6^^,^^14^^,^^24^

In recent years, the scientific community has recognized urgency as a priority patient-reported outcome and key treatment target for IBD patients. The guidelines of the American College of Gastroenterology and international recommendations for clinical trials have begun to include control of urgency as a treatment objective and an efficacy endpoint.^2^^,^^4^ In parallel, the pharmaceutical industry has included bowel urgency as a secondary outcome in registrational studies of new drugs, such as Janus kinase inhibitors (upadacitinib) and S1P receptor modulators (etrasimod), demonstrating rapid and clinically significant improvements.^25^^,^^26^

Despite these advances, standardized measurement of urgency remains a challenge. Traditional clinical indices, such as the Mayo score or the Patient-Reported Outcomes-2 (PRO-2), do not include a specific assessment of urgency. In contrast, historical scales, such as the Simple Clinical Colitis Activity Index (SCCAI), devote only a single generic item to this symptom.^6^ In recent years, more targeted instruments have been developed. The best known is the Urgency Numeric Rating Scale (NRS), a single 11-point item initially validated in UC^2^^,^^27^^,^^28^ and subsequently adapted and validated in CD.^10^ This scale has shown excellent test-retest reliability (intraclass correlation coefficient >0.80) and construct validity, demonstrating sensitivity to clinical changes and patients’ perceptions.^10^^,^^29^ However, the Urgency NRS is limited to a unidimensional assessment of severity, without considering frequency, psychosocial impact, coping strategies, and emotional correlates.

The qualitative literature confirms the need for a broader assessment. Patient interviews have highlighted that the experience of urgency is not limited to the intensity of the need, but includes anticipatory anxiety, constant planning of daily movements, social limitations, and fear of accidents.^29^^,^^30^ Moreover, many patients consider even modest reductions (1-3 points) in the Urgency NRS meaningful, suggesting that perceptions of improvement are multidimensional.^29^ Unlike diarrhea and rectal bleeding, which are included in all clinical indices, urgency has no gold-standard measure, hindering comparative assessment across studies and the design of targeted clinical trials.

Considering these elements, a precise knowledge and operational gap emerge. There is a lack of a multidimensional, IBD-specific instrument capable of capturing the whole experience of bowel urgency, including emotional, behavioral, and quality-of-life factors. A dedicated questionnaire developed in accordance with the Food and Drug Administration guidelines for patient-reported outcomes (PROs) and psychometrically validated could fill this gap and serve as a key endpoint in clinical trials and in everyday practice. This paper aimed to clarify the construct of bowel urgency and provide a measurement framework for clinical and research applications.

Specifically, this concept analysis aimed to provide 3 advances beyond existing narrative reviews. First, we derive an integrated set of defining attributes that connect the sensorimotor core of urgency with its psychosocial and behavioral “persistence layer” in everyday life. Second, we delineate the conceptual boundaries between urgency and related anorectal symptoms (tenesmus, fecal incontinence, and increased stool frequency) using clinical and patient-reported examples. Third, we propose a preliminary multidimensional measurement blueprint for bowel urgency, outlining key domains and empirical referents to inform development of an IBD-specific patient-reported outcome suitable for both routine care and clinical trials.

## Methods

We approached the analysis with an explicitly narrative stance, using Walker and Avant’s 8 steps as both scaffold and storyline. Walker and Avant’s methodological process follows few steps, in order to (1) select a concept; (2) determine the aims or purposes of the analysis; (3) identify all uses of the concept that can be discovered; (4) determine the defining attributes; (5) identify a model case, (6) identify borderline, related, contrary, invented, and illegitimate cases; (7) identify antecedents and consequences; and (8) define empirical referents. We aimed to keep the rigor of a structured concept analysis, seeking to articulate a clear conceptual and operational definition of bowel urgency, while preserving the voices and contexts that make urgency a lived phenomenon.

### Steps 1 and 2: Selecting the concept and specifying aims

We selected bowel urgency for its salience to patients and its underrepresentation in indices and endpoints. Our primary aim was to refine the meaning of urgency (attributes, antecedents, consequences) and to translate this meaning into measurement guidance suitable for clinical practice and trials. A secondary aim was to retain and integrate the existing evidence base, epidemiology, qualitative accounts, physiology, and therapeutic effects, without discarding any citations from the foundational narrative review.^1^^,^^2^^,^^4–6^^,^^10^^,^^11^^,^^14^^,^^15^^,^^21^^,^^25^^,^^29^^,^^31–40^

### Step 3: Identifying uses of the concept

To detail the uses of bowel/rectal/fecal urgency, we first clarified the general-language semantics of urgency and then mapped its biomedical/clinical usage and patient-centered lexicon. In everyday English, urgency denotes something “very important and needing attention immediately,” foregrounding time pressure and immediacy; major dictionaries also highlight shades of insistence/pressure/urge (eg, Merriam-Webster). These lexical cues anticipate core experiential elements later seen in clinical contexts (immediacy, difficulty deferring).^41^ Controlled vocabularies converge on a compact definition. The International Continence Society defines fecal/rectal urgency as the complaint of a sudden, compelling desire to defecate that is difficult to defer. International Continence Society also names fecal urgency warning time as the interval from first sensation to defecation or incontinence, capturing the temporal core of the construct. SNOMED CT/MedGen/HPO registers the concept and its synonyms (defecation urgency; urgent desire for stool; precipitancy of defecation), supporting interoperability.^42^

Clinical and guideline-style sources^43^ consistently separate urgency from tenesmus. Tenesmus is a “painful/ineffective urge with little or no stool despite an empty rectum.” In contrast, urgency stresses a sudden need that is hard to defer and often connotes risk of incontinence, and the two are listed side by side (and not synonymously) in government and specialty resources for IBD/proctitis. This distinction is vital for measurement and communication.

### Evidence base and search frame

We narratively integrated literature published between January 2016 and March 2025 and indexed in MEDLINE (via PubMed), PsycINFO (via Ovid), Scopus, and Web of Science. A 3-step search strategy was adopted. First, a preliminary search of MEDLINE and PsycINFO was conducted to identify relevant keywords and indexing terms related to bowel urgency in IBD. Second, these terms were systematically combined and adapted to the syntax of each database, integrating symptom-related terms (eg, “bowel urgency,” “fecal urgency,” “rectal urgency”), disease labels (eg, “inflammatory bowel disease,” “ulcerative colitis,” “Crohn disease”), and measurement-related terms (eg, “patient-reported outcome,” “numeric rating scale,” “PRO instrument”) (Supplemental File 1). Finally, the reference lists of all included studies were manually screened to identify additional eligible articles. The complete MEDLINE search strategy and the corresponding core term combinations for PsycINFO, Scopus, and Web of Science are reported in the Supplemental File. We included peer-reviewed studies in adults with IBD that reported prevalence, phenomenology, mechanistic correlates, prognostic value, measurement, or treatment effects on bowel urgency. We excluded case reports, pediatric-only samples, non-IBD populations, and studies that mentioned urgency as an eligibility criterion without further description. Screening was conducted by IC and VV, with DN as the third ­reviewer. Additional records were identified by citation chasing from key studies and recent reviews. The study selection process is summarized in a PRISMA-ScR flow diagram (Supplemental File).

### Step 4: Deriving defining attributes

Defining attributes were identified through iterative, constant comparison across quantitative and qualitative sources. For each study, we extracted language and findings describing the phenomenology, severity gradients, and functional impact of bowel urgency. Attributes were retained (1) when they recurred across different study designs and populations and (2) when their absence transformed the experience into a recognizably different construct (eg, tenesmus, high stool frequency without time pressure).^2^^,^^6^^,^^29^ We maintained an attribute-evidence matrix linking each candidate attribute to supporting extracts and citations; the mapping is provided in Supplemental File. Candidate attributes were then refined through discussion within the multidisciplinary author team.

### Steps 5 and 6: Constructing model, related, borderline, and contrary cases

Guided by the candidate attributes, we developed brief clinical vignettes reflecting model, related, borderline, and contrary cases of bowel urgency. Each vignette was iteratively mapped back to the attributes to ensure internal consistency and to stress test the construct’s boundaries. Cases were revised until there was consensus that the model case exemplified the full constellation of attributes. In contrast, related and borderline cases captured partial or overlapping phenomenology but did not meet the full definition of urgency.

### Step 7: Mapping antecedents and consequences

We organized antecedents along biological (inflammation, tissue mechanics, sensorimotor and pelvic floor physiology, barrier/microbiome) and psychosocial (anxiety, vigilance) axes, and consequences from patient to system levels. We privileged findings with longitudinal associations or prognostic value.^1^^,^^4^^,^^6^^,^^11^^,^^14^^,^^31^

### Step 8: Specifying empirical referents

For empirical referents, we catalogued existing urgency-related items and scales (eg, Urgency NRS; legacy indices such as SCCAI; absence from PRO-2/Mayo) and examined the extent to which they covered the proposed attributes. We then outlined potential domains and example item formulations for a multidimensional urgency PRO, explicitly linking each domain to the underlying attributes and to existing regulatory guidance on PRO development.^2^^,^^29^^,^^40^

### Synthesis approach and handling of heterogeneity

Because definitions, instruments, and reporting practices vary widely, we adopted a narrative, integrative synthesis rather than a quantitative analysis. We used an abductive approach: preliminary interpretations were grounded in observed patterns in the data, then iteratively refined in light of emerging theoretical and clinical insights. Where feasible, we compared urgency-specific findings with related constructs (eg, stool frequency, rectal bleeding, pain) to clarify conceptual boundaries. Heterogeneity in study design, inclusion criteria, and outcome measures was handled by (1) grouping studies according to their primary focus (eg, epidemiology, mechanistic correlates, clinical outcomes, qualitative experience, measurement) and (2) prioritizing consistency of direction and clinical plausibility over pooled estimates. This approach allowed us to respect contextual nuances while still drawing cross-study inferences about defining attributes, antecedents, consequences, and empirical referents.^1^^,^^10^^,^^29^^,^^34^

### Rigor, reflexivity, and limitations of method

To enhance rigor, we implemented dual independent screening and data extraction, maintained a detailed audit trail of inclusion decisions, and aimed to preserve source fidelity by retaining the original wording of key concepts wherever possible. Reflexivity was addressed by explicitly acknowledging, within the author team, pre-existing clinical and research commitments regarding the importance of urgency in IBD, and by periodically revisiting whether these assumptions were unduly shaping interpretation. Nonetheless, the evidence base is heterogeneous and often relies on single-item urgency measures, with limited longitudinal and mechanistic data. Therefore, some steps of Walker and Avant’s framework, particularly case construction and empirical referents, necessarily draw on theoretically informed extrapolation rather than on direct evidence alone. As a result, our conclusions prioritize conceptual clarity and clinical interpretability over quantitative precision and should be read accordingly.^40^

We attempted to reduce selection bias via dual screening/abstraction and to preserve source fidelity by retaining all citations from the base review. Nonetheless, as a concept analysis nested within a narrative review, our work is limited by heterogeneity in definitions (“urgency” phrasing), the lack of standardized cutoffs, and the predominance of data from Europe/North America. These choices favor breadth and transferability over pooled estimates and should be read accordingly.

## Uses and definitions of the concept

In IBD, “bowel/rectal/fecal urgency” denotes a sudden and compelling need to defecate that is difficult or impossible to defer, often accompanied by risk of incontinence.^2^ Clinically, it reflects increased rectal drive; in research, it serves as an outcome sensitive to therapeutic change; and in patients’ lives, it acts as an organizing force that dictates vigilance and daily planning.^29^ While urgency may also occur in functional bowel disorders, its expression in IBD is distinguished by its association with mucosal inflammation, prognostic relevance, and persistence even during clinical or endoscopic remission.^2^^,^^11^ Bowel urgency should be clearly differentiated from tenesmus, which refers to a persistent or false sensation of the need to defecate, even when the rectum is empty. In the reviewed literature, bowel urgency is used in at least 3 distinct but overlapping ways. First, in everyday language, patients use “urgency” to describe episodes in which they feel they must rush to the toilet, often emphasizing the emotional experience of panic, embarrassment, or fear of an accident. Second, in clinical practice, urgency functions as a triage signal and a marker of incomplete disease control: clinicians use it to identify patients at risk of incontinence, to guide treatment escalation, and to assess whether remission is meaningful from the patient’s perspective. Third, in research and clinical trials, urgency increasingly appears as a secondary or exploratory endpoint, in which changes in urgency may diverge from changes in stool frequency and rectal bleeding, suggesting a partly independent symptom trajectory. Together, these contextual uses illustrate how bowel urgency operates simultaneously as a lived experience, a ­clinical signal, and a research outcome, underscoring the need for a precise, operational definition that bridges lay language, clinical reasoning, and measurement in trials.

### Conceptual boundaries with related constructs

Bowel urgency needs to be distinguished from several related anorectal symptoms. In this analysis, bowel urgency denotes a sudden and compelling need to defecate that is difficult or impossible to defer, usually accompanied by perceived risk of incontinence. Tenesmus refers to a persistent or recurrent sensation of the need to defecate despite little or no stool in the rectum; time pressure may be absent, but the feeling of incomplete evacuation predominates. Fecal incontinence is the involuntary loss of stool or gas, which may occur with or without preceding urgency. Increased stool frequency describes a higher-than-usual number of bowel movements and can occur with preserved deferability and without fear of accidents.

In practice, a patient who passes 5 loose stools per day but can comfortably postpone defecation for 20 to 30 minutes while at work would be better characterized by increased stool frequency than by bowel urgency. Conversely, a patient who has only 1 or 2 bowel movements per day but experiences sudden, nondeferrable urges with near-misses or occasional leakage exemplifies bowel urgency rather than frequency. These contrasts underscore that time pressure, perceived controllability, and risk of incontinence—rather than stool count alone—are central to the construct of bowel urgency.

### Defining attributes

Evidence converges on 7 key attributes, as shown in Table 1.

**Table 1.** Defining attributes of bowel urgency, clinical implications, and component layers.

Attribute	Description	Clinical implication	Component layer
**Sudden onset**	Urgency begins abruptly and without warning, often escalating within seconds and interrupting normal activities. The unpredictability generates loss of confidence and fear of accidents.^1^ ^,^ ^2^	Clinicians should inquire about sudden urges even during remission phases, as unpredictability increases anxiety and social withdrawal.	Sensorimotor core
**Perceived uncontrollability/inability to defer**	Patients experience an overwhelming conviction that deferral is impossible, even when continence is maintained. This sense of loss of control drives avoidance and immediate action.^2^ ^,^ ^37^	Assess perceived control and coping strategies; include questions on confidence in managing urges during clinical interviews.	Sensorimotor core
**Compressed time to toilet**	The available window to reach the restroom is drastically shortened. Patients plan routes based on restroom proximity and constantly evaluate risk of not arriving in time.^11^ ^,^ ^29^	Evaluate accessibility of restrooms and encourage safety planning (eg, identifying facilities, workplace accommodations).	Sensorimotor core
**Fear/risk of incontinence**	A pervasive fear of leakage or public embarrassment dominates the experience. Prior near misses increase vigilance and muscular tension.^11^ ^,^ ^15^ ^,^ ^29^	Screen for anticipatory fear and embarrassment; integrate relaxation or cognitive-behavioral techniques to reduce hypervigilance.	Sensorimotor core
**Anticipatory anxiety and hypervigilance**	Even in remission, patients maintain a constant alertness to bodily sensations and restroom locations. Anxiety lowers perception thresholds and perpetuates symptoms.^29^ ^,^ ^34^ ^,^ ^37^	Recognize urgency as a biopsychosocial phenomenon; consider referral to psychological support when hypervigilance is evident.	Psychosocial persistence layer
**Behavioral planning and avoidance**	Patients modify diets, travel routes, and social activities to manage uncertainty. Over time, this leads to social restriction and decreased quality of life.^6^ ^,^ ^11^ ^,^ ^14^	Ask about lifestyle adaptations; address avoidance behaviors with education, exposure, or tailored coping plans.	Psychosocial persistence layer
**Persistence despite inflammatory quiescence**	Urgency persists despite endoscopic or clinical remission due to rectal hypersensitivity, dyssynergia, or learned alarm responses.^1^ ^,^ ^10^ ^,^ ^32^ ^,^ ^34^	Differentiate inflammatory from sensorimotor-driven urgency and refer for anorectal or pelvic floor assessment when appropriate.	Psychosocial persistence layer

Alt text: Defining Attributes of Bowel Urgency, Clinical Implications, and Component Layers.

Their interaction defines bowel urgency as both a measurable clinical construct and a lived, organizing experience.

### Antecedents

The experience of bowel urgency arises from the intersection of biological, sensorimotor, and psychosocial mechanisms. These antecedents act in concert, sometimes sequentially, sometimes persistently, creating the physiological substrate and emotional context in which urgency is felt and sustained.

Distal inflammation, particularly in the rectum, sets the stage for urgency. Proctitis, ulceration, and mucosal edema alter the mechanical properties of the rectal wall, reducing its compliance and amplifying sensory input.^1^^,^^4^^,^^31^ Even after mucosal healing, residual fibrosis or submucosal stiffening can persist, preserving heightened sensation and creating a “primed” rectum that overreacts to regular filling. Patients often describe the signal as disproportionate—“I feel full when there’s almost nothing there,” reflecting the biomechanical legacy of prior ­inflammation.

Altered communication among the rectum, the pelvic floor, and the central nervous system also shapes urgency. Lower ­distension thresholds, increased phasic contractions, and episodes of ­pelvic floor dyssynergia or spasm create a mismatch between rectal sensation and motor control.^10^^,^^32^^,^^35^ These sensorimotor abnormalities translate biological signals into an exaggerated urge, sometimes occurring even in the absence of visible inflammation. The rectum becomes both overly sensitive and functionally disorganized—ready to contract before it should, responding to stimuli that once went unnoticed.

The intestinal barrier and microbial ecosystem modulate the interface between inflammation and sensation. Loss of epithelial integrity, coupled with postinflammatory neuromodulation, enhances exposure of mucosal nerves to luminal stimuli.^1^^,^^4^^,^^37^ Dysbiosis may further alter motility and visceral perception through microbial metabolites and immune signaling. In this sense, urgency can be seen as a neuroimmune dialogue gone awry, a hypersensitive gut reacting to subtle shifts in microbial or epithelial tone.

Beyond the gut, central processes of perception and emotion amplify urgency. Anxiety, stress reactivity, and catastrophizing heighten interoceptive focus and lower the threshold for alarm.^34^^,^^37^^,^^44^ Patients often learn to scan continuously for danger signals, whether bodily sensations or environmental risks, creating a feedback loop where vigilance intensifies perception and perception sustains vigilance. This coupling of feeling and emotion transforms urgency from a momentary signal into a chronic state of readiness.

Clinical circumstances shape how these mechanisms manifest. Distal disease distribution increases exposure of the rectum to inflammatory and mechanical stress. Active flares accentuate urgency through combined inflammatory and sensory pathways, while postoperative changes, such as altered anatomy or reduced reservoir function, can perpetuate the symptom even in remission.^10^^,^^11^^,^^15^ Incomplete therapeutic response or delayed mucosal healing can prolong urgency despite apparent clinical improvement, illustrating how biological and experiential recovery may diverge.

### Consequences

**Figure 1:**
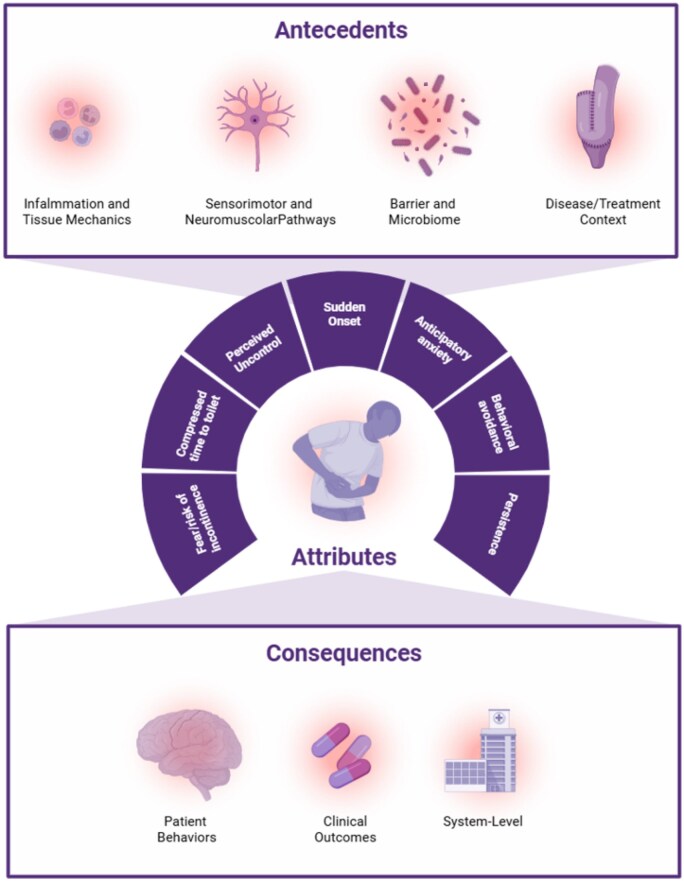
Conceptual model of bowel urgency in inflammatory bowel disease. Bowel urgency arises from interacting biological and psychosocial mechanisms. Antecedents include inflammation and tissue mechanics, sensorimotor and neuromuscular pathways, alterations in barriers and the microbiome, and disease or treatment context. These pathways converge into 7 defining attributes—ranging from sudden onset and perceived uncontrollability to anticipatory anxiety, behavioral avoidance, and persistence beyond inflammation. The resulting consequences span patient behaviors, clinical outcomes, and system-level impacts.

The consequences of bowel urgency unfold across multiple layers, from intimate personal distress to measurable system-level burdens. What begins as a physiological symptom reverberates through emotional, relational, occupational, and clinical ­domains, shaping both the lived experience of IBD and its broader healthcare implications (Figure 1**)**.

At the individual level, bowel urgency exerts a deep psychological and emotional toll. Fear of accidents and public embarrassment becomes a constant backdrop to daily life, fueling anticipatory anxiety and hypervigilance.^29^^,^^37^ Many patients withdraw from social interactions, limit travel, or avoid situations in which immediate restroom access is uncertain. Intimate relationships may be affected by the need for constant control and the fear of unpredictability. Sleep is often fragmented, patients report awakening to perceived urges or remaining hyperalert through the night. Over time, chronic fatigue, mood disturbance, and reduced confidence in bodily reliability erode overall health-related quality of life, even when objective disease activity appears minimal.

### Role function

Urgency also intrudes into the domains of work and productivity. Episodes can lead to absenteeism during flares but more commonly manifest as presenteeism, being physically present yet distracted or constrained by symptom vigilance.^14^^,^^15^ Routine tasks, meetings, and commutes may require constant planning or compromise. The result is a subtle but persistent reduction in functional capacity and career advancement opportunities. In some cases, the stigma surrounding urgency can discourage disclosure or workplace accommodation, reinforcing isolation and a loss of agency.

From a clinical standpoint, urgency serves not only as a symptom, but also as a prognostic signal. Patients with high or persistent urgency have higher odds of hospitalization, corticosteroid exposure, and even colectomy within a year, independent of stool frequency or bleeding.^6^^,^^15^^,^^45^^,^^46^ This dissociation underscores that urgency reflects dimensions of disease activity and sensorimotor dysfunction not fully captured by standard indices. Its persistence may therefore indicate an incomplete response to therapy or unresolved physiological sensitization, warranting closer clinical attention.

At the health-system level, bowel urgency is associated with increased healthcare utilization, including more frequent visits, urgent consultations, and medication adjustments.^10^^,^^11^ Yet despite its measurable burden, urgency remains underrecognized in disability assessments and quality registries. This invisibility translates into missed opportunities for intervention and policy recognition. Reframing urgency as a quantifiable, modifiable determinant of IBD-related disability could align patient-centered outcomes with system efficiency, bridging the gap between what patients report and what systems record.

### Empirical referents (measurement)

No validated multidimensional, IBD-specific urgency PRO integrating intensity, frequency, deferability, time to toilet, leakage/near misses, psychosocial impact, coping/avoidance, and predictability/triggers.

The Urgency NRS is among the most commonly used measures. It consists of a single 0 to 10 item assessing urgency severity over the previous 24 hours. This tool has demonstrated strong test-retest reliability, solid construct validity, and good responsiveness in both UC and CD.^2^^,^^10^^,^^33^

Among legacy indices, only the SCCAI includes a specific item on urgency, whereas the Mayo Score and PRO-2 lack an explicit assessment of this symptom.^6^

Moreover, qualitative research, through patient interviews and symptom diaries, has revealed key experiential domains such as anticipatory anxiety, planning and avoidance behaviors, and coping mechanisms that are not captured by existing single-item measures.^1^^,^^29^^,^^30^

### Case exemplars

**Figure 2:**
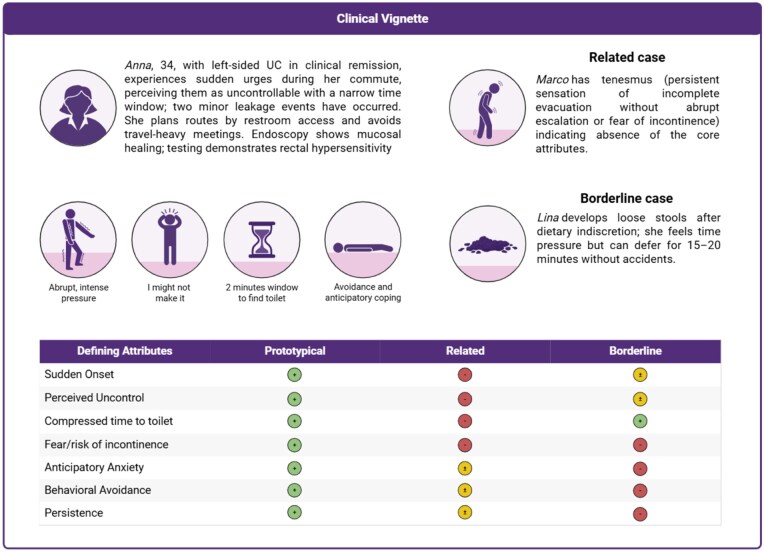
The model case (Anna) demonstrates the whole constellation of urgency attributes: sudden onset, perceived uncontrollability, compressed time to toilet, and anticipatory coping, persisting despite mucosal healing and reflecting rectal hypersensitivity. The related case (Marco) exemplifies tenesmus—persistent sensation of incomplete evacuation without abrupt escalation or fear of incontinence—indicating absence of the core attributes. The borderline case (Lina) depicts transient urgency with partial deferability, distinguishing situational urgency from the persistent, multidimensional phenomenon characteristic of inflammatory bowel disease. UC, ulcerative colitis.

In a model case (all attributes present), Anna, 34 years of age, with left-sided UC in clinical remission, experiences sudden ­urges during her commute, perceiving them as uncontrollable with a narrow time window; 2 minor leakage events have occurred. She plans routes by restroom access and avoids travel-heavy meetings. Endoscopy shows mucosal healing; testing demonstrates rectal hypersensitivity.^2^ Contrary case (attributes absent). In another case, Paolo passes one formed stool daily without suddenness, time pressure, or avoidance behaviors. Related case (overlapping but distinct) and borderline case (partial attributes) are displayed in Figure 2.

The related case depicts a patient with frequent loose stools driven predominantly by dietary indiscretion, who reports inconvenience but minimal time pressure, little anticipatory anxiety, and no fear of accidents; here, increased frequency is present but bowel urgency, as defined in this analysis, is not. The borderline case portrays a patient with intermittent episodes of sudden urgency during flares but preserved deferability and limited behavioral modification between flares, illustrating how partial combinations of attributes approach, but do not fully meet, the construct of bowel urgency.

## Discussion

This concept analysis invites a continuous reading of urgency, from the seconds that define an episode to the systems that shape a life. The sensorimotor core (suddenness, uncontrollability, time pressure, incontinence risk) intersects with a psychosocial halo (anticipation, vigilance, planning/avoidance) that accrues over time. This layering explains 3 recurrent clinical puzzles: why urgency can persist despite mucosal healing, why it predicts outcomes independent of other symptoms, and why single-item measures can be both valuable and insufficient.^1^^,^^2^^,^^6^

This concept analysis adds to previous narrative reviews in 3 main ways. First, it articulates a coherent set of defining attributes that link the sensorimotor core of bowel urgency with its psychosocial and behavioral consequences in everyday life. Second, it draws explicit conceptual boundaries between urgency and related anorectal symptoms—tenesmus, fecal incontinence, and increased stool frequency—using clinical and patient-reported examples. Third, it translates these attributes into a preliminary multidimensional measurement blueprint, specifying domains and empirical referents for an IBD-specific urgency PRO. Together, these contributions move the field beyond viewing urgency as a single checklist item and towards a more granular, patient-centered construct.

The “so what” of this analysis is both clinical and methodological. Clinically, the proposed attributes and cases offer a structured lens for deciding when to prioritize anti-inflammatory escalation, when to investigate anorectal function, and when to target psychological or behavioral mechanisms that perpetuate urgency despite quiescent inflammation. Methodologically, the measurement blueprint provides a starting point for developing and validating urgency instruments that can be incorporated as prespecified endpoints in clinical trials and observational cohorts. This may enable more precise phenotyping, better alignment between clinician- and patient-defined remission, and ultimately more tailored treatment strategies.

### Clinical translation: An agenda for everyday care

First, ask—and document—urgency explicitly. A 3-question screen (NRS intensity; “Could you defer?”; “How many minutes to a toilet?”) quickly surfaces risk. For persistent urgency in quiescent disease, move beyond inflammation: assess rectal sensation and compliance and screen for pelvic floor dyssynergia.^31^^,^^32^^,^^35^ Pair pharmacologic optimization with behavioral interventions (urgency-suppression skills, exposure to feared contexts) and clear safety plans (eg, workplace accommodations). Given the link to mood and participation, integrate psychological support, recognizing the bidirectional relations highlighted by Gracie et al.,^34^^,^^44^ Nigam et al.,^37^ and Jairath et al.^29^

### Mechanism-informed phenotypes

Our synthesis suggests at least 2 working phenotypes: inflammation-dominant urgency, in which symptom improvement tracks mucosal healing, and sensorimotor-dominant urgency, in which rectal hypersensitivity and pelvic floor ­dysfunction sustain episodes despite quiescence.^1^^,^^4^^,^^10^ Identifying the phenotype at the bedside can guide whether to prioritize anti-inflammatory escalation, anorectal rehabilitation, or psychological therapies.^32^^,^^34^

### Measurement and the case for a multidimensional PRO

The Urgency NRS remains an efficient barometer of intensity and is responsive to therapies.^10^^,^^33^ Yet to align with the whole construct, assessment should also include frequency, deferability, time to toilet, leakage, anxiety/vigilance, and behavioral impact, the 8 domains proposed here. Psychometric development should pursue both brief screens for clinical use and adaptive digital formats for trials, with the minimally significant difference anchored to within-patient change that patients recognize as meaningful.^15^^,^^29^^,^^30^

Legacy indices such as SCCAI or PRO-2 insufficiently capture these dimensions, underscoring the need for an urgency-specific IBD PRO.^2^^,^^15^^,^^47^

### Trial design and regulatory relevance

Urgency has emerged as a sensitive endpoint across modern therapies: JAK inhibitors show early improvements (often within week 1), S1P receptor modulators demonstrate clinically meaningful gains by week 12, and anti-IL-23 agents yield sustained reductions.^9^^,^^25^^,^^26^^,^^33^^,^^36^^,^^48^

Future trials should prespecify urgency outcomes, report weekly trajectories, and define composite responders (eg, ≥2-point NRS reduction plus improved deferability or time-to-toilet). Such designs elevate patient-valued benefits alongside endoscopic healing and align with regulatory expectations for symptom-based endpoints.^49^

### Health-system considerations

Because urgency drives utilization and costs while remaining underrecognized,^11^^,^^14^^,^^15^ health systems should embed urgency screening in routine IBD assessment and registries. Integrating patient-reported data with endoscopy, biomarkers, and anorectal metrics would enable learning health feedback loops, helping identify which phenotypes benefit from which interventions and when.^21^

Such data could inform reimbursement policies and resource allocation, reframing urgency as a measurable and modifiable component of IBD disability.

### Strengths and limitations

Strengths include the integration of qualitative and quantitative evidence, preservation of the existing citation base, and an explicit measurement blueprint. This concept analysis also has important limitations. The available evidence is heterogeneous and often restricted to single-item measures of urgency, with limited longitudinal and mechanistic data. As a result, several aspects of Walker and Avant’s framework—particularly the refinement of cases and empirical referents—required theoretically informed extrapolation beyond the direct data. In addition, our synthesis is anchored in studies of adults with IBD and may not fully capture how bowel urgency is conceptualized in pediatric populations or in other chronic bowel conditions. These limitations mean that our proposed attributes and measurement blueprint should be regarded as a structured starting point, rather than a definitive, final model.

### Future directions

Priorities include (1) validating the proposed multidimensional urgency PRO measure across UC and CD; (2) mapping biological correlates (healing, hypersensitivity, pelvic floor function, microbiome) to PRO trajectories; (3) testing targeted adjuncts (pelvic floor biofeedback, neuromodulation, psychological interventions) for sensorimotor-dominant urgency; and (4) embedding urgency endpoints into phase 3 programs and real-world registries to align treat-to-target strategies with what patients feel first and last.

## Conclusion

Bowel urgency in IBD is a symptom that organizes lives and predicts outcomes. Clarifying its attributes, antecedents, and consequences reveals where current measurement falls short and where clinical pathways can change. Building and validating a multidimensional urgency PRO, while continuing to assess and treat sensorimotor and psychosocial contributors, offers a practical route to care that is both mechanistically informed and unmistakably patient centered.

## Supplementary Material

izag018_Supplementary_Data

## Data Availability

No data are available for this manuscript.
