# Supplementary material for: Bowel urgency in inflammatory bowel disease: A concept analysis
Source: Inflamm Bowel Dis. 2026 Feb 25;32(8):1600–11. doi: 10.1093/ibd/izag018 (PMC13414550; doi:10.1093/ibd/izag018)

**Search strategy**

| **PSYCINFO (Via Ovid)**  ((“bowel urgency” OR “fecal urgency” OR “faecal urgency” OR “rectal urgency” OR “defecation urgency” OR “urgency to defecate” OR “need to rush” OR “inability to defer” OR “cannot defer” OR (urgency AND (bowel OR stool OR defecat*))) AND (“inflammatory bowel disease” OR IBD OR “ulcerative colitis” OR “crohn disease” OR “crohn’s disease” OR (colitis AND (ulcerative OR chronic))) AND (“patient-reported outcome” OR “patient reported outcome” OR PRO OR PROs OR “PRO instrument” OR “patient-reported measure” OR “patient-centered measure” OR “symptom score” OR “symptom assessment” OR “symptom reporting” OR “urgency score” OR “urgency assessment” OR “urgency rating” OR “numeric rating scale” OR NRS OR “visual analogue scale” OR VAS OR diary OR “patient diary” OR “symptom diary” OR “patient-generated” OR “self-reported” OR “electronic PRO” OR ePRO OR “ecological momentary assessment” OR EMA OR questionnaire OR instrument* OR measure*))  N= 421 |
| --- |
| **WEB OF SCIENCE (Via Clarivate)**  ((“bowel urgency” OR “fecal urgency” OR “faecal urgency” OR “rectal urgency” OR “defecation urgency” OR “urgency to defecate” OR “need to rush” OR “inability to defer” OR “cannot defer” OR (urgency AND (bowel OR stool OR defecat*))) AND (“inflammatory bowel disease” OR IBD OR “ulcerative colitis” OR “crohn disease” OR “crohn’s disease” OR (colitis AND (ulcerative OR chronic))) AND (“patient-reported outcome” OR “patient reported outcome” OR PRO OR PROs OR “PRO instrument” OR “patient-reported measure” OR “patient-centered measure” OR “symptom score” OR “symptom assessment” OR “symptom reporting” OR “urgency score” OR “urgency assessment” OR “urgency rating” OR “numeric rating scale” OR NRS OR “visual analogue scale” OR VAS OR diary OR “patient diary” OR “symptom diary” OR “patient-generated” OR “self-reported” OR “electronic PRO” OR ePRO OR “ecological momentary assessment” OR EMA OR questionnaire OR instrument* OR measure*))  N= 444 |
| **MEDLINE (via Pubmed)**  (“bowel urgency”[tiab] OR “fecal urgency”[tiab] OR “faecal urgency”[tiab] OR “rectal urgency”[tiab] OR “defecation urgency”[tiab] OR “urgency to defecate”[tiab] OR “need to rush”[tiab] OR “inability to defer”[tiab] OR “cannot defer”[tiab] OR (urgency[tiab] AND (bowel[tiab] OR stool[tiab] OR defecat*[tiab]))) AND (“Inflammatory Bowel Diseases”[Mesh] OR “Crohn Disease”[Mesh] OR “ulcerative colitis”[Mesh] OR “inflammatory bowel disease”[tiab] OR IBD[tiab] OR “crohn disease”[tiab] OR “crohn’s disease”[tiab] OR (“colitis”[tiab] AND (ulcerative[tiab] OR chronic[tiab]))) AND (“patient-reported outcome”[tiab] OR “patient reported outcome”[tiab] OR PRO[tiab] OR PROs[tiab] OR “PRO instrument”[tiab] OR “patient-reported measure”[tiab] OR “patient-centered measure”[tiab] OR “symptom score”[tiab] OR “symptom assessment”[tiab] OR “symptom reporting”[tiab] OR “urgency score”[tiab] OR “urgency assessment”[tiab] OR “urgency rating”[tiab] OR “numeric rating scale”[tiab] OR NRS[tiab] OR “visual analogue scale”[tiab] OR VAS[tiab] OR diary[tiab] OR “patient diary”[tiab] OR “symptom diary”[tiab] OR “patient-generated”[tiab] OR “self-reported”[tiab] OR “electronic PRO”[tiab] OR ePRO[tiab] OR “ecological momentary assessment”[tiab] OR EMA[tiab] OR questionnaire[tiab] OR instrument*[tiab] OR measure*[tiab])  N= 215 |
| **SCOPUS**  ((“bowel urgency” OR “fecal urgency” OR “faecal urgency” OR “rectal urgency” OR “defecation urgency” OR “urgency to defecate” OR “need to rush” OR “inability to defer” OR “cannot defer” OR (urgency AND (bowel OR stool OR defecat*))) AND (“inflammatory bowel disease” OR IBD OR “ulcerative colitis” OR “crohn disease” OR “crohn’s disease” OR (colitis AND (ulcerative OR chronic))) AND (“patient-reported outcome” OR “patient reported outcome” OR PRO OR PROs OR “PRO instrument” OR “patient-reported measure” OR “patient-centered measure” OR “symptom score” OR “symptom assessment” OR “symptom reporting” OR “urgency score” OR “urgency assessment” OR “urgency rating” OR “numeric rating scale” OR NRS OR “visual analogue scale” OR VAS OR diary OR “patient diary” OR “symptom diary” OR “patient-generated” OR “self-reported” OR “electronic PRO” OR ePRO OR “ecological momentary assessment” OR EMA OR questionnaire OR instrument* OR measure*))  N= 260 |

Note: Searches were limited to studies published between January 2016 and March 2025. Search strategies were adapted to each database's syntax. No restrictions on study design were applied at the search stage.

**Attribute–evidence mapping matrix supporting the seven defining attributes of bowel urgency in IBD.**

Legend: ✓ indicates that the source explicitly discusses or assesses the attribute (definition, patient report, measurement item, or outcome framing). Attributes correspond to Table 1 in the main manuscript.

| **Study (first author, year)** | **Study focus / design** | **Urgency definition or measure** | **A1 Sudden onset** | **A2 Uncontrollability** | **A3 Time-to-toilet** | **A4 Incontinence fear** | **A5 Anticipatory anxiety** | **A6 Planning/avoidance** | **A7 Persistence in remission** | **Key** |
| --- | --- | --- | --- | --- | --- | --- | --- | --- | --- | --- |
| Ghosh et al., 2021 | Clinical trial | SCCAI |  |  |  | ✓ |  |  | ✓ | Assesses urgency using SCCAI. Links urgency to fear of accidents/leakage. Notes that urgency can persist despite clinical/endoscopic improvement. |
| Dubinsky (a) et al., 2022 | Clinical trial | Urgency Numeric Rating Scale | ✓ | ✓ | ✓ | ✓ | ✓ | ✓ | ✓ | Assesses urgency using Urgency Numeric Rating Scale. Frames urgency as difficulty delaying defecation with a limited time window. Links urgency to fear of accidents/leakage. Describes planning/avoidance behaviours (e.g., toilet access, activity restriction). Notes anticipatory worry/hypervigilance related to possible urgency episodes. Notes that urgency can persist… |
| Dubinsky (b) et al., 2022 | Clinical trial | Urgency Numeric Rating Scale |  | ✓ |  |  |  | ✓ | ✓ | Assesses urgency using Urgency Numeric Rating Scale. Emphasises difficulty delaying defecation (loss of control). Describes planning/avoidance behaviours (e.g., toilet access, activity restriction). Notes that urgency can persist despite clinical/endoscopic improvement. |
| Dubinsky et al., 2022 | Clinical trial | Urgency Numeric Rating Scale | ✓ | ✓ | ✓ | ✓ | ✓ | ✓ | ✓ | Assesses urgency using Urgency Numeric Rating Scale. Frames urgency as difficulty delaying defecation with a limited time window. Links urgency to fear of accidents/leakage. Describes planning/avoidance behaviours (e.g., toilet access, activity restriction). Notes anticipatory worry/hypervigilance related to possible urgency episodes. Notes that urgency can persist… |
| Caron et al., 2023 | Clinical trial | Urgency Numeric Rating Scale | ✓ | ✓ | ✓ | ✓ | ✓ | ✓ | ✓ | Assesses urgency using Urgency Numeric Rating Scale. Frames urgency as difficulty delaying defecation with a limited time window. Links urgency to fear of accidents/leakage. Describes planning/avoidance behaviours (e.g., toilet access, activity restriction). Notes anticipatory worry/hypervigilance related to possible urgency episodes. Notes that urgency can persist… |
| Danese et al., 2023 | Clinical trial | Urgency Numeric Rating Scale | ✓ | ✓ |  |  | ✓ | ✓ | ✓ | Assesses urgency using Urgency Numeric Rating Scale. Emphasises difficulty delaying defecation (loss of control). Describes planning/avoidance behaviours (e.g., toilet access, activity restriction). Notes anticipatory worry/hypervigilance related to possible urgency episodes. Notes that urgency can persist despite clinical/endoscopic improvement. |
| Dubinsky et al., 2023 | Clinical trial | Urgency Numeric Rating Scale | ✓ | ✓ | ✓ | ✓ | ✓ | ✓ | ✓ | Assesses urgency using Urgency Numeric Rating Scale. Frames urgency as difficulty delaying defecation with a limited time window. Links urgency to fear of accidents/leakage. Describes planning/avoidance behaviours (e.g., toilet access, activity restriction). Notes anticipatory worry/hypervigilance related to possible urgency episodes. Notes that urgency can persist… |
| Dubinsky et al., 2023 | Measurement development/validation | Urgency Numeric Rating Scale | ✓ | ✓ | ✓ | ✓ | ✓ | ✓ | ✓ | Assesses urgency using Urgency Numeric Rating Scale. Frames urgency as difficulty delaying defecation with a limited time window. Links urgency to fear of accidents/leakage. Describes planning/avoidance behaviours (e.g., toilet access, activity restriction). Notes anticipatory worry/hypervigilance related to possible urgency episodes. Notes that urgency can persist… |
| Loftus et al., 2023 | Clinical trial | VAS |  | ✓ |  |  |  | ✓ | ✓ | Assesses urgency using VAS. Emphasises difficulty delaying defecation (loss of control). Describes planning/avoidance behaviours (e.g., toilet access, activity restriction). Notes that urgency can persist despite clinical/endoscopic improvement. |
| Pakpoor et al., 2023 | Measurement development/validation | Urgency Numeric Rating Scale | ✓ | ✓ | ✓ | ✓ | ✓ | ✓ | ✓ | Assesses urgency using Urgency Numeric Rating Scale. Frames urgency as difficulty delaying defecation with a limited time window. Links urgency to fear of accidents/leakage. Describes planning/avoidance behaviours (e.g., toilet access, activity restriction). Notes anticipatory worry/hypervigilance related to possible urgency episodes. Notes that urgency can persist… |
| Sninsky et al., 2023 | Clinical trial | SCCAI |  | ✓ | ✓ | ✓ | ✓ |  | ✓ | Assesses urgency using SCCAI. Frames urgency as difficulty delaying defecation with a limited time window. Links urgency to fear of accidents/leakage. Notes anticipatory worry/hypervigilance related to possible urgency episodes. Notes that urgency can persist despite clinical/endoscopic improvement. |
| Wol et al., 2023 | Measurement development/validation | SCCAI | ✓ |  |  | ✓ | ✓ | ✓ | ✓ | Assesses urgency using SCCAI. Links urgency to fear of accidents/leakage. Describes planning/avoidance behaviours (e.g., toilet access, activity restriction). Notes anticipatory worry/hypervigilance related to possible urgency episodes. Notes that urgency can persist despite clinical/endoscopic improvement. |
| Atreya et al., 2024 | Clinical trial | VAS |  | ✓ |  |  |  | ✓ | ✓ | Assesses urgency using VAS. Emphasises difficulty delaying defecation (loss of control). Describes planning/avoidance behaviours (e.g., toilet access, activity restriction). Notes that urgency can persist despite clinical/endoscopic improvement. |
| Atreya et al., 2024 | Clinical trial | Urgency Numeric Rating Scale | ✓ | ✓ |  | ✓ | ✓ | ✓ | ✓ | Assesses urgency using Urgency Numeric Rating Scale. Emphasises difficulty delaying defecation (loss of control). Links urgency to fear of accidents/leakage. Describes planning/avoidance behaviours (e.g., toilet access, activity restriction). Notes anticipatory worry/hypervigilance related to possible urgency episodes. Notes that urgency can persist despite clinical/endoscopic improvement. |
| Gibble et al., 2024 | Clinical trial | Urgency Numeric Rating Scale | ✓ | ✓ | ✓ | ✓ | ✓ | ✓ | ✓ | Assesses urgency using Urgency Numeric Rating Scale. Frames urgency as difficulty delaying defecation with a limited time window. Links urgency to fear of accidents/leakage. Describes planning/avoidance behaviours (e.g., toilet access, activity restriction). Notes anticipatory worry/hypervigilance related to possible urgency episodes. Notes that urgency can persist… |
| Lewis et al., 2024 | Consensus/Delphi | Not specified | ✓ | ✓ | ✓ | ✓ | ✓ |  | ✓ | Addresses bowel urgency as a clinical symptom/endpoint. Frames urgency as difficulty delaying defecation with a limited time window. Links urgency to fear of accidents/leakage. Notes anticipatory worry/hypervigilance related to possible urgency episodes. Notes that urgency can persist despite clinical/endoscopic improvement. |
| Nigam et al., 2024 | Survey/cross-sectional | VAS | ✓ | ✓ |  |  | ✓ |  | ✓ | Assesses urgency using VAS. Emphasises difficulty delaying defecation (loss of control). Notes anticipatory worry/hypervigilance related to possible urgency episodes. Notes that urgency can persist despite clinical/endoscopic improvement. |
| Peyrin-Biroulet et al., 2024 | Clinical trial | Urgency Numeric Rating Scale |  | ✓ |  | ✓ |  |  | ✓ | Assesses urgency using Urgency Numeric Rating Scale. Emphasises difficulty delaying defecation (loss of control). Links urgency to fear of accidents/leakage. Notes that urgency can persist despite clinical/endoscopic improvement. |
| Wespi et al., 2024 | Survey/cross-sectional | VAS | ✓ |  |  | ✓ |  | ✓ | ✓ | Assesses urgency using VAS. Links urgency to fear of accidents/leakage. Describes planning/avoidance behaviours (e.g., toilet access, activity restriction). Notes that urgency can persist despite clinical/endoscopic improvement. |
| Carboni et al., 2025 | Clinical trial | SCCAI | ✓ | ✓ |  | ✓ |  | ✓ | ✓ | Assesses urgency using SCCAI. Emphasises difficulty delaying defecation (loss of control). Links urgency to fear of accidents/leakage. Describes planning/avoidance behaviours (e.g., toilet access, activity restriction). Notes that urgency can persist despite clinical/endoscopic improvement. |
| Chaparro et al., 2025 | Clinical trial | Urgency Numeric Rating Scale | ✓ | ✓ |  |  |  | ✓ | ✓ | Assesses urgency using Urgency Numeric Rating Scale. Emphasises difficulty delaying defecation (loss of control). Describes planning/avoidance behaviours (e.g., toilet access, activity restriction). Notes that urgency can persist despite clinical/endoscopic improvement. |
| Clemow et al., 2025 | Clinical trial | Urgency Numeric Rating Scale | ✓ | ✓ | ✓ | ✓ |  | ✓ | ✓ | Assesses urgency using Urgency Numeric Rating Scale. Frames urgency as difficulty delaying defecation with a limited time window. Links urgency to fear of accidents/leakage. Describes planning/avoidance behaviours (e.g., toilet access, activity restriction). Notes that urgency can persist despite clinical/endoscopic improvement. |
| Marín-Jiménez et al., 2025 | Consensus/Delphi | VAS | ✓ | ✓ | ✓ |  | ✓ |  | ✓ | Assesses urgency using VAS. Frames urgency as difficulty delaying defecation with a limited time window. Notes anticipatory worry/hypervigilance related to possible urgency episodes. Notes that urgency can persist despite clinical/endoscopic improvement. |
| **No. of sources supporting attribute** |  |  | **17** | **20** | **11** | **16** | **14** | **17** | **23** | **Total sources: 23** |

**PRISMA-ScR flow diagram**
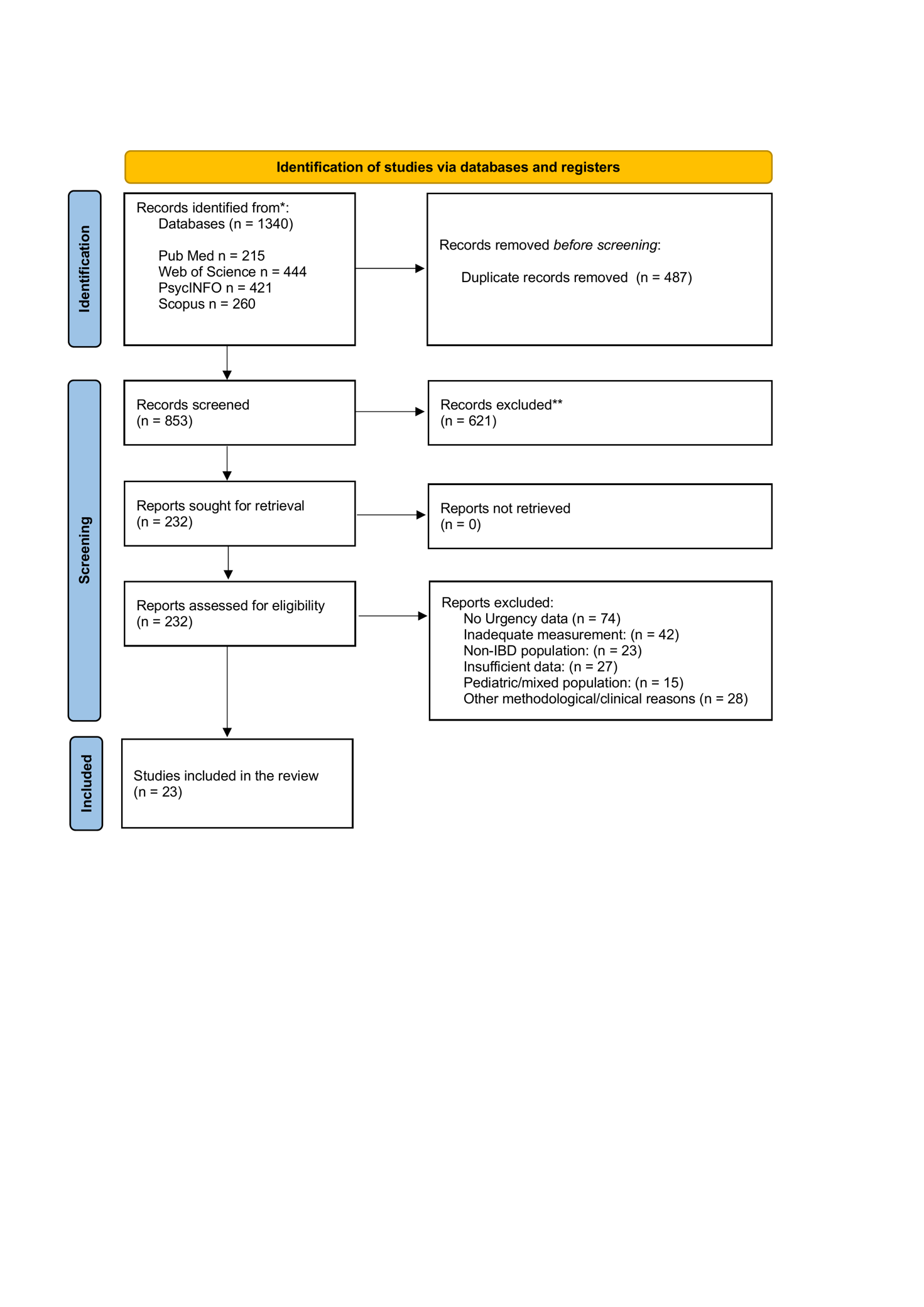

Supplement: izag018_Supplementary_Data [file izag018_supplementary_data.docx]
